# SUR1-E1506K mutation impairs glucose tolerance and promotes vulnerable atherosclerotic plaque phenotype in hypercholesterolemic mice

**DOI:** 10.1371/journal.pone.0258408

**Published:** 2021-11-12

**Authors:** Erika Gurzeler, Anna-Kaisa Ruotsalainen, Anssi Laine, Teemu Valkama, Sanna Kettunen, Markku Laakso, Seppo Ylä-Herttuala

**Affiliations:** 1 A.I. Virtanen Institute, University of Eastern Finland, Kuopio, Finland; 2 Department of Medicine, University of Eastern Finland and Kuopio University Hospital, Kuopio, Finland; 3 Gene Therapy Unit, Kuopio University Hospital, Kuopio, Finland; 4 Heart Center, Kuopio University Hospital, Kuopio, Finland; Max Delbruck Centrum fur Molekulare Medizin Berlin Buch, GERMANY

## Abstract

**Background and aims:**

Diabetes is a major risk factor of atherosclerosis and its complications. The loss-of-function mutation E1506K in the sulfonylurea receptor 1 (SUR1-E1506K) induces hyperinsulinemia in infancy, leading to impaired glucose tolerance and increased risk of type 2 diabetes. In this study, we investigate the effect of SUR1-E1506K mutation on atherogenesis in hypercholesterolemic LDLR^-/-^ mice.

**Methods:**

SUR1-E1506K mutated mice were cross-bred with LDLR^-/-^ mice (SUR1Δ/LDLR^-/-^), 6 months old mice were fed a western-diet (WD) for 6 months to induce advanced atherosclerotic plaques. At the age of 12 months, atherosclerosis and plaque morphology were analyzed and mRNA gene expression were measured from aortic sections and macrophages. Glucose metabolism was characterized before and after WD. Results were compared to age-matched LDLR^-/-^ mice.

**Results:**

Advanced atherosclerotic plaques did not differ in size between the two strains. However, in SUR1Δ/LDLR^-/-^ mice, plaque necrotic area was increased and smooth muscle cell number was reduced, resulting in higher plaque vulnerability index in SUR1Δ/LDLR^-/-^ mice compared to LDLR^-/-^ mice. SUR1Δ/LDLR^-/-^ mice exhibited impaired glucose tolerance and elevated fasting glucose after WD. The positive staining area of IL-1β and NLRP3 inflammasome were increased in aortic sections in SUR1Δ/LDLR^-/-^ mice compared to LDLR^-/-^ mice, and IL-18 plasma level was elevated in SUR1Δ/LDLR^-/-^ mice. Finally, the mRNA expression of IL-1β and IL-18 were increased in SUR1Δ/LDLR^-/-^ bone marrow derived macrophages in comparison to LDLR^-/-^ macrophages in response to LPS.

**Conclusions:**

SUR1-E1506K mutation impairs glucose tolerance and increases arterial inflammation, which promotes a vulnerable atherosclerotic plaque phenotype in LDLR^-/-^ mice.

## Introduction

Type 2 diabetes mellitus and its complications are increasing worldwide, leading to death and reduced quality of life in a large number of patients [1, 2]. Both pre-diabetes and diabetes have been associated with cardiovascular diseases and are known to increase the risk of myocardial infarction, stroke and peripheral arterial disease [3, 4]. Atherosclerosis is a chronic inflammatory disease, defined by accumulation of lipids, lipoproteins, smooth muscle cells, connective tissue and inflammatory cells in the intimal layer of the arterial wall causing the formation of atherosclerotic plaques [5]. Over time, those plaques promote arterial wall stiffening and narrowing of arterial lumen, causing reduced blood flow and plaque rupture resulting in ischemic events [6]. Early atherosclerosis develops through LDL retention and its modifications in the intima [7], attracting macrophages that engulf modified LDL and form foam cells and fatty streaks [8]. This accumulation of lipids and macrophage foam cells provoke local vascular cytokine secretion and increase the expression of chemotactic proteins and adhesion molecules, thus promoting accumulation of inflammatory cells [9, 10]. Enhanced pro-inflammatory stimuli within the plaques promote smooth muscle cell apoptosis and reduce the synthesis and secretion of extracellular matrix components that destabilize a fibrous cap on top of the atherosclerotic plaque, turning it into a more vulnerable and rupture-prone phenotype [10]. In diabetes, insulin resistance, defective insulin signalling and hyperglycemia are key driving factors of atherosclerotic plaque development, independently of conventional risk factors, like hypertension and hyperlipidemia [11, 12]. Diabetes increases vascular oxidative stress and the secretion of pro-inflammatory cytokines that lead to endothelial dysfunction and increased adhesion and activation of leukocytes in the vascular wall, promoting atherogenesis [13].

The Sulfonylurea Receptor- 1 (SUR1) and the inward rectifier K^+^ channel KIR6.2 (KCNJ11) form ATP-sensitive potassium (K_ATP_) channels, which are the main regulators of insulin secretion in response to glucose concentration and ATP [14]. During low plasma glucose levels, the K_ATP_ channels are open and the pancreatic β-cell membrane is hyperpolarized, preventing insulin secretion. In response to an increase in glucose and ATP levels the K_ATP_ channels are closed leading to depolarization of the cell membrane and the release of insulin. Loss-of-function mutations in either Kir6.2 or SUR1 cause a permanent membrane depolarization in β-cells, leading to persistent Ca^2+^ influx, and thus continuous insulin release. Mutations of both genes have been linked with congenital hyperinsulinemia and type 2 diabetes [15]. Carriers of the specific E1506K loss-of-function mutation in SUR1 have mild congenital hyperinsulinism and are at high risk of developing type 2 diabetes later in life [14, 16].

Diabetes has a pivotal role in the development and maturation of atherosclerotic plaques. Therefore, a better understanding of the specific mechanisms on how diabetes affects atherogenesis is urgently needed to develop new therapeutic strategies for the treatment of atherosclerotic vascular diseases in diabetes. In this study we hypothesized that the development of diabetic condition during aging in SUR1Δ/LDLR^-/-^ mice would promote atherosclerosis. For that, we crossed SUR1-E1506K (SUR1) mice [16] to hyperlipidemic mice lacking the low-density lipoprotein receptor (LDLR^-/-^) [17]. In this mouse model, we elucidate the atherosclerotic plaque development and plaque phenotype during impaired glucose metabolism conditions. Our results show that SUR1Δ/LDLR^-/-^ mice exhibited elevated fasting glucose and impaired glucose tolerance, with no changes in plasma lipid levels or atherosclerotic lesion area. However, atherosclerotic plaques showed a more vulnerable phenotype in SUR1Δ/LDLR^-/-^ mice by increased plaque necrosis and reduced number of smooth muscle cells in aortic plaques in comparison to LDLR^-/-^ mice. Moreover, the expression of pro-inflammatory cytokine IL-1β and NLRP3 inflammasome were increased in aortic sections in SUR1Δ/LDLR^-/-^ mice, suggesting that impaired glucose metabolism promotes plaque vulnerability via increased arterial wall inflammation in SUR1Δ/LDLR^-/-^ mice.

## Materials and methods

### Mice

SUR1-E1506K mutated mice (C57Bl/6J background) [16] were crossbred with LDLR^-/-^ mice (The Jackson Laboratory) and backcrossed for ten generations. Male SUR1Δ/LDLR^-/-^ mice were used. Age-matched LDLR^-/-^ mice were used as controls. The mice were fed a chow diet (Teklad Global 16% Protein Rodent Diet: 12% of calories from fat and 0% cholesterol) until age of 6 months and then fed western diet (WD) (TD88137, Harlan Teklad: 42% of calories from fat and 0.15% cholesterol) for additional 6 months. When mice were 12 months old the study was terminated by sacrificing the mice with CO_2_ and tissue and plasma samples were taken. The mice were housed in the Animal Centre of the University of Eastern Finland in controlled conditions for temperature and humidity, using a 12 h light/dark cycle and had an *ad libitum* access to food and water. All animal procedures were approved by the Animal Experiment Board in Finland and carried out according to the guidelines of the Finnish Act on Animal Experimentation and Experimental Animal Committee of the University of Eastern Finland.

### Metabolic analyses

Prior to intraperitoneal glucose tolerance test (IPGTT) the mice were fasted for 3 h. 1g/kg glucose was injected i.p. and blood glucose was measured from tail vein at baseline, 15, 30, 60 and 120 min after injection. Intraperitoneal insulin tolerance test (IPITT) was performed on fed mice, during the test food and water were removed from the cages. 0.25IU/kg of insulin (Actrapid^®^ Penfill^®^, Novo Nordisk A/s) was injected i.p. and blood glucose was measured from tail vein at baseline, 15, 30, 45 and 90 min after injection.

Three hours after food removal, blood was withdrawn from saphenous vein and fasting glucose was measured with a Glucometer (Ascensia Elite XL, Bayer). Plasma insulin levels were measured with an Ultra-sensitive mouse insulin ELISA kit (#90080, Chrystal Chem.). Plasma lipid levels were measured from 12 months old mice by a veterinary diagnostic laboratory Movet Oy (Kuopio, Finland).

### Histology

Mice were sacrificed at the age of 12 months after feeding WD for 6 months and tissues were perfused with phosphate buffered saline (Dulbecco’s Phosphate Buffered Saline, D8537), followed by 6 hours fixation in 4% paraformaldehyde (pH 7.4) in 7.5% sucrose. For analysis of atherosclerosis, aortic root and brachiocephalic arteries were embedded in paraffin for serial cross-sectional analysis (5 μm sections, mean of 3 sections per mouse at 25 μm intervals). The average plaque area was quantified from hematoxylin-eosin stained cross-sections of the aortic root and brachiocephalic artery with Image J 1.48V Software. For the analysis of aortic root plaque morphology, aortic plaque necrosis was measured from hematoxylin-eosin stained sections by morphological criteria in which plaque acellular areas were regarded as necrotic areas. In addition, aortic root plaque macrophages were immunostained with mMQ antibody (anti-mouse macrophage, mMQ AIA31240, Accurate Chemical & Scientific Corp.), smooth muscle cells with a αSMA antibody (anti-alpha smooth muscle actin, ab15267, Abcam) and aortic adventitial microvessels with a CD31 antibody (BC-CM303A, Biocare Medical). All histological images were taken with Nikon Eclipse microscope with a Ds-Ri2 camera (Nikon Instruments Europe BV). For analysis of IL-1β, NLRP3, MCP-1 and CD68 expression in aortic plaques, aortic sections were stained with IL-1β (ab9722, Abcam), NLRP3 (ab214185, Abcam), MCP-1 (Ab7202, Abcam), and CD68 (ab125212, Abcam) primary antibodies. To analyze plaque calcification and collagen content, aortic sections were stained with Alizarin Red S and Sirius Red S, respectively. Finally, macrophage area, necrotic area, collagen content and smooth muscle cell content were used to measure plaque vulnerability index from aortic root plaques as follows: (macrophages-% + necrotic area-%)/(collagen-% + smooth muscle cells-%) = vulnerability index [18–20]. To quantify fibrous cap thickness from aortic plaques, three serial sections per mouse were stained with Hematoxylin-eosin staining, and three to five of the thinnest caps were measured and the average was calculated. All quantifications of plaque morphology were done in relation to total plaque area with Image J 1.48V Software based on threshold. All analyses were done in a blinded manner.

### Isolation and differentiation of bone marrow derived macrophages

SUR1Δ/LDLR^-/-^ and LDLR^-/-^ mice were euthanized and femoral and tibial bones were dissected. Bone marrow (BM) was expelled by flushing with ice cold PBS. Cells were differentiated for 7 days in RPMI1640 medium with 10% fetal bovine serum (FBS), 1% Penicillin-Streptomycin (P/S) and 20 ng/ml macrophage colony stimulating factor (M-CSF, Miltenyi Biotec^®^) on 10 cm Petri dishes, at 37°C. Resulting bone marrow derived macrophages (BMDMs) were plated in 12 well plate (800 000 cells/well) and treated with To measure the expression of pro-inflammatory cytokines, the macrophages were treated with LPS (10 ng/ml) and oxLDL (50 μg/ml).

### Gene expression analysis

Total RNA of BMDMs was isolated with Trizol reagent (RNA/DNA/Protein Isolation Reagent, Molecular Research Center Inc.) and mRNA expression levels were measured with quantitative RT-PCR (StepOnePlus, Applied Biosystems). The following Assays–on demand were used: IL-1β (Mm.PT.58.42940223), Nlrp3 (Mm.PT.58.13974318) and IL-18 (Mm.PT.58.42776691) (IDT, Integrated DNA Technologies, Inc.). The expression levels were normalized GAPDH (Mm.PT.39a1, IDT, Integrated DNA Technologies, Inc) expression in the same tissue or Tbp (Mm.PT.39a22214839) for oxLDL treated BMDM. IL-18 plasma levels were measure with a quantitative ELISA kit for mouse IL-18 (Mouse IL-18 ELISA kit, MBL).

### Statistics

GraphPad Prism5 was used for statistical analysis. If two groups were compared at different timepoints two-way ANOVA, followed by Bonferroni-test was used. If more than two groups were compared one-way ANOVA, followed by Bonferroni-test was applied. Otherwise, Student’s t-test was applied. Data are shown as mean ± SEM. P < 0.05 was considered as statistically significant. The following symbols are used in figures and tables: * p < 0.05, ** p <0.01, *** p < 0.001.

## Results

### SUR1-E1506K mutation has no effect on atherosclerotic plaque size, but promotes plaque maturation in LDLR^-/-^ mice

**Fig 1:**
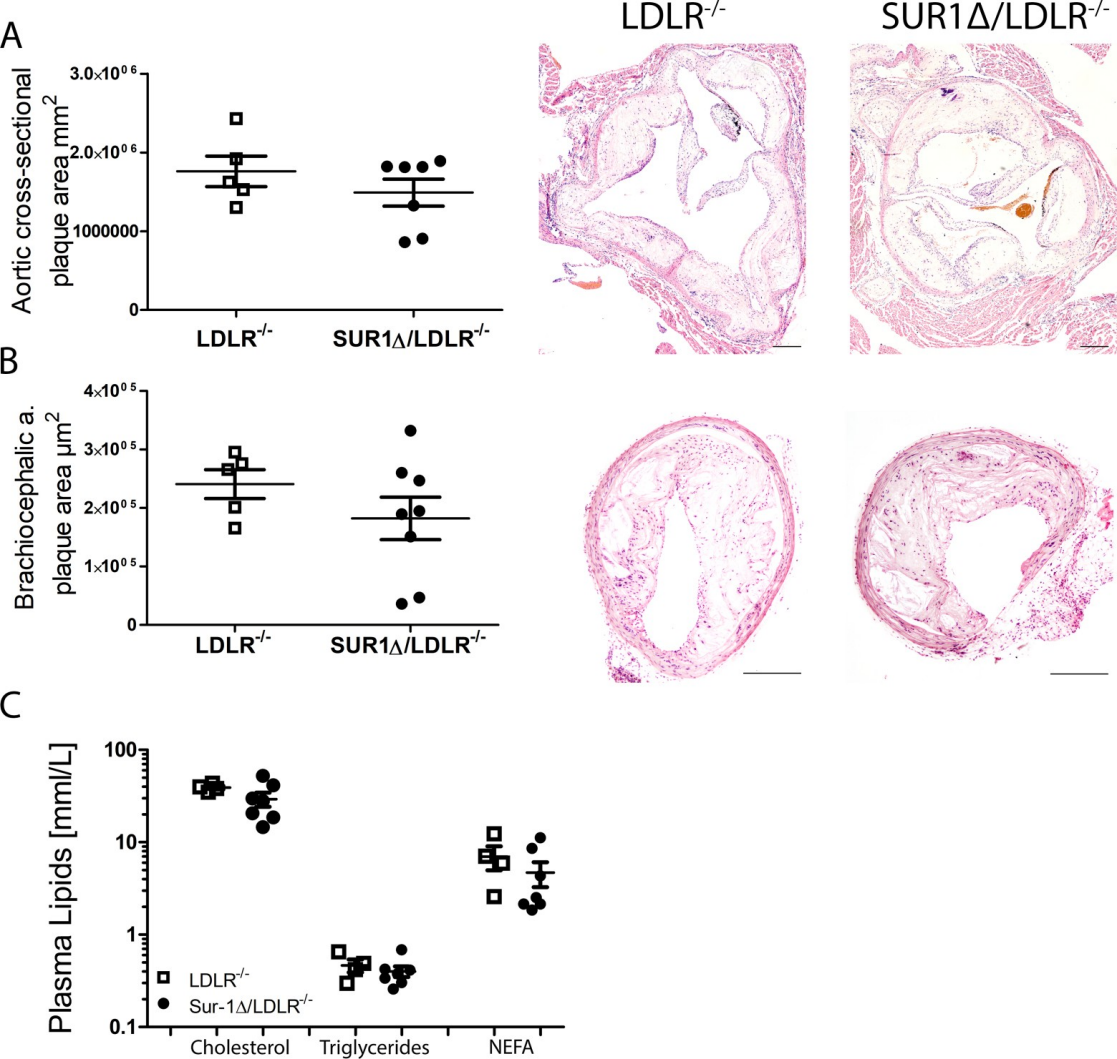
SUR1Δ/LDLR^-/-^ mice do not show differences in atherosclerotic plaque size or in plasma lipid levels when compared to LDLR^-/-^ mice. (A) Hematoxylin-eosin stained cross-sectional plaque area in the aortic root of SUR1Δ/LDLR^-/-^ (n = 7) mice compared to LDLR^-/-^ (n = 5) mice, (B) Plaque area in brachiocephalic artery in SUR1Δ/LDLR^-/-^ (n = 8) and LDLR^-/-^ (n = 5) mice, (C) Plasma levels of cholesterol, triglycerides and NEFA at the age of 12 months in SUR1Δ/LDLR^-/-^ (n = 7) and LDLR^-/-^ (n = 4) mice. Scale bar: 200μm. Data are shown as mean ± SEM and Student’s t-test was used for statistical analysis.

To investigate the effect of impaired glucose metabolism on atherosclerosis development, plaque burden and morphology were analyzed at the aortic root and in the brachiocephalic artery at age of 12 months after 6 months WD. To induce advanced atherosclerotic plaques animals were fed a WD for 6 month [17]. No difference was detected in total plaque area between these two strains, neither in the aortic root (Fig 1A) nor in the brachiocephalic artery (Fig 1B). Furthermore, plasma lipid levels such as cholesterol, triglycerides or non-esterified free fatty acids (NEFA) did not change between SUR1Δ/LDLR^-/-^ and LDLR^-/-^ mice after 6 months of WD (Fig 1C).

**Fig 2:**
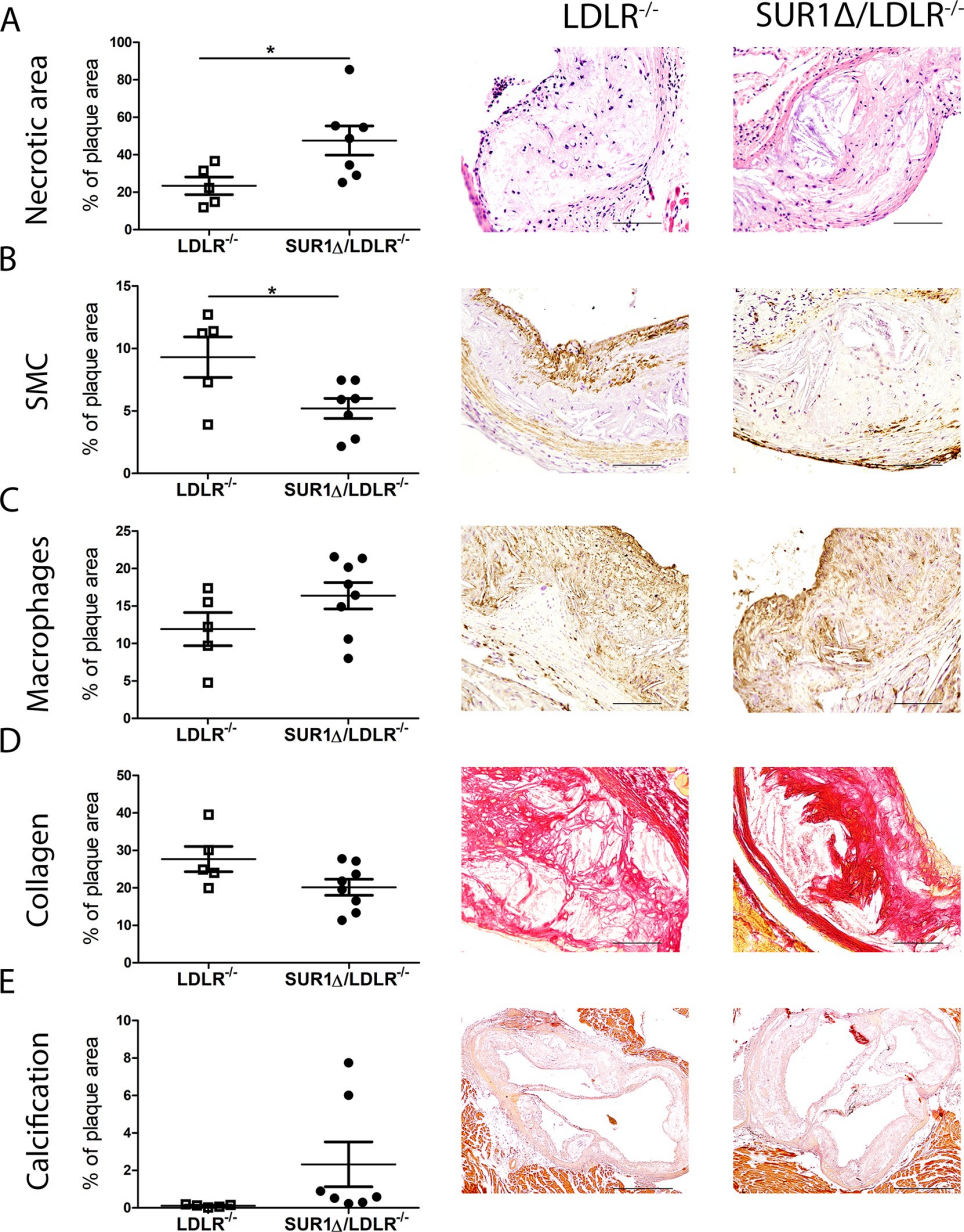
Increased plaque necrotic area and reduced plaque smooth muscle cells in SUR1Δ/LDLR^-/-^ (n = 7–8) compared to LDLR^-/-^ (n = 5) mice. (A) Necrotic area in plaques measured from hematoxylin-eosin staining, scale bar: 100 μm, (B) Smooth muscle content in plaques measured with αSMA staining, scale bar: 100μm, (C) Macrophage positive area measured by mMQ staining, scale bar: 100μm, (D) Collagen content in plaques, measured by Sirius red staining, scale bar: 100μm, (E) Calcified plaque area measured by Alzarin Red S staining, scale bar: 500μm. Data are shown as mean ± SEM and Student’s t-test was used for statistical analysis. * p < 0.05.

Interestingly, we found that SUR1Δ/LDLR^-/-^ mice had significantly increased necrotic core area in comparison to LDLR^-/-^ mice (Fig 2A). Additionally, we could show that in plaques of SUR1Δ/LDLR^-/-^ mice, smooth muscle cells amount was significantly reduced compared to LDLR^-/-^ mice (Fig 2B). No change in macrophage number or plaque collagen content could be seen between the SUR1Δ/LDLR^-/-^ and LDLR^-/-^ mice (Fig 2C and 2D). Also, no difference in calcification was detected between SUR1Δ/LDLR^-/-^ mice and LDLR^-/-^ controls (Fig 2E) and overall, only two SUR1Δ/LDLR^-/-^ mice had noteworthy calcified lesions in the aorta (Fig 2E).

### Increased plaque vulnerability in SUR1-E1506K mutated LDLR^-/-^ mice

**Fig 3:**
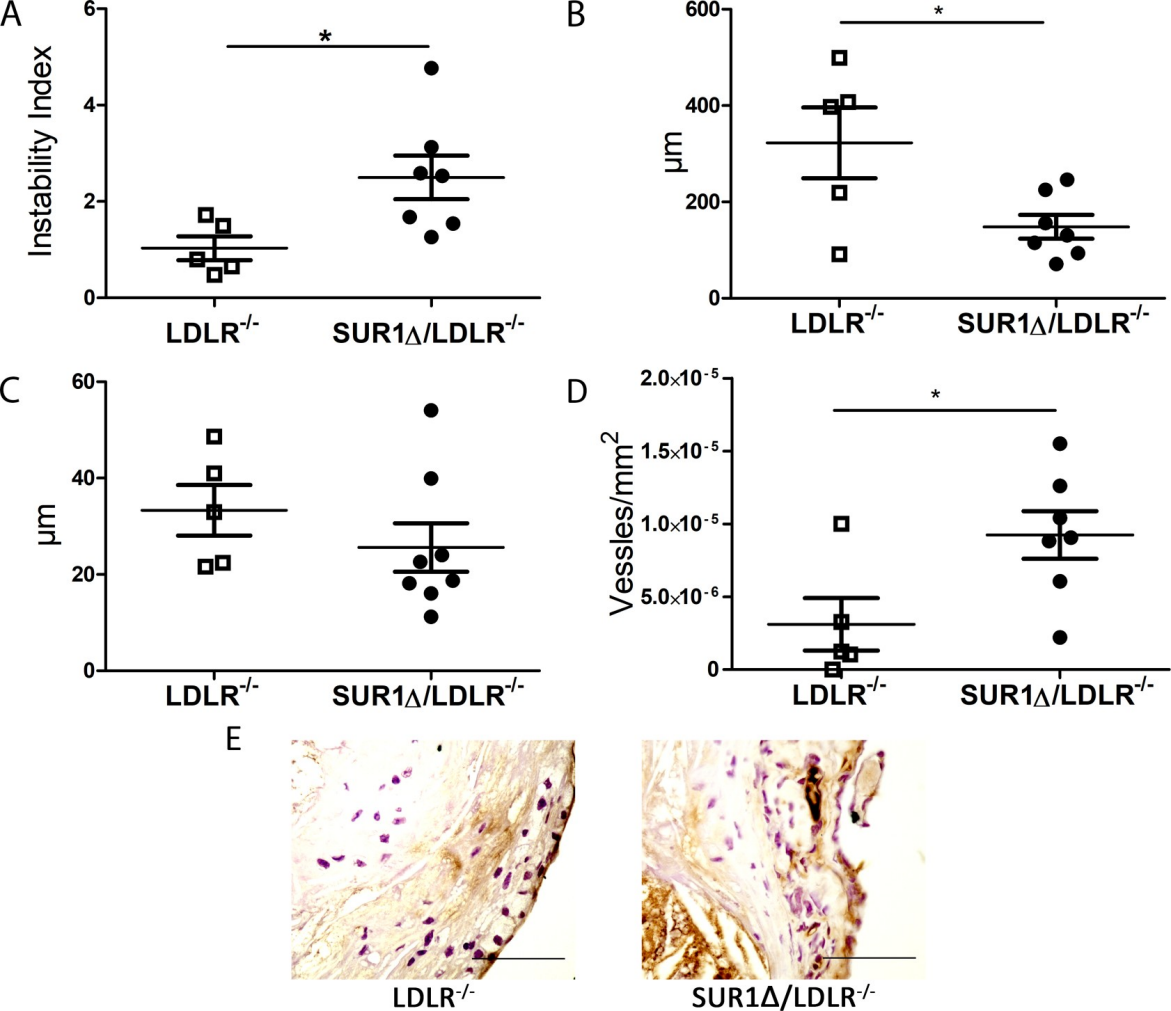
SUR1Δ/LDLR^-/-^ (n = 7) mice have increased plaque instability index and increased plaque microvessels compared to LDLR^-/-^ (n = 5) mice. (A) Instability index calculated as the ratio between instability parameters such as macrophage content and necrotic area and stability parameters, including collagen content and smooth muscle cells, (B) Fibrous cap thickness in the brachiocephalic artery, (C) Fibrous cap thickness in the aortic valves, (D) SUR1Δ/LDLR^-/-^ mice showed increase adventitial microvessel area in CD31 immunohistological staining, (E) Representative pictures of CD31 staining. Scale bar: 50μm (n = 5–7). Data are shown as mean ± SEM and Student’s t-test was used for statistical analysis. * p < 0.05.

To assess the overall stability of the plaques, the vulnerability index and fibrotic cap thickness were determined. The vulnerability index was calculated as a ratio between unstable features, comprising of macrophage content and necrotic area and stable features, including collagen content and smooth muscle cells. The vulnerability index was 2.5-fold higher in SUR1Δ/LDLR^-/-^ mice compared to LDLR^-/-^ mice (Fig 3A). In addition, the fibrotic cap thickness in the brachiocephalic artery and aortic valves were reduced in SUR1Δ/LDLR^-/-^ mice in comparison to LDLR^-/-^ mice (Fig 3B and 3C). Increased instability index together with the reduced cap thickness suggest a more vulnerable plaque phenotype in SUR1Δ/LDLR^-/-^ mice in comparison to LDLR^-/-^ mice.

Another hallmark of advanced and vulnerable plaques in humans is intraplaque neovascularization [13]. Earlier studies have shown that the amount of microvessels correlateswith progression of atherosclerosis and plaque vulnerability [10]. In this study, we found that SUR1Δ/LDLR^-/-^ mice have more aortic adventitial microvessels compared to LDLR^-/-^ mice (Fig 3D), which is in line with a landscape of more mature plaques and increased plaque vulnerability index in SUR1Δ/LDLR^-/-^ mice. Nevertheless, no intraplaque hemorrhages were seen in the aortic root or brachiocephalic artery plaques.

### SUR1-E1506K mutation increases fasting glucose level and impairs glucose tolerance in LDLR^-/-^ mice

**Fig 4:**
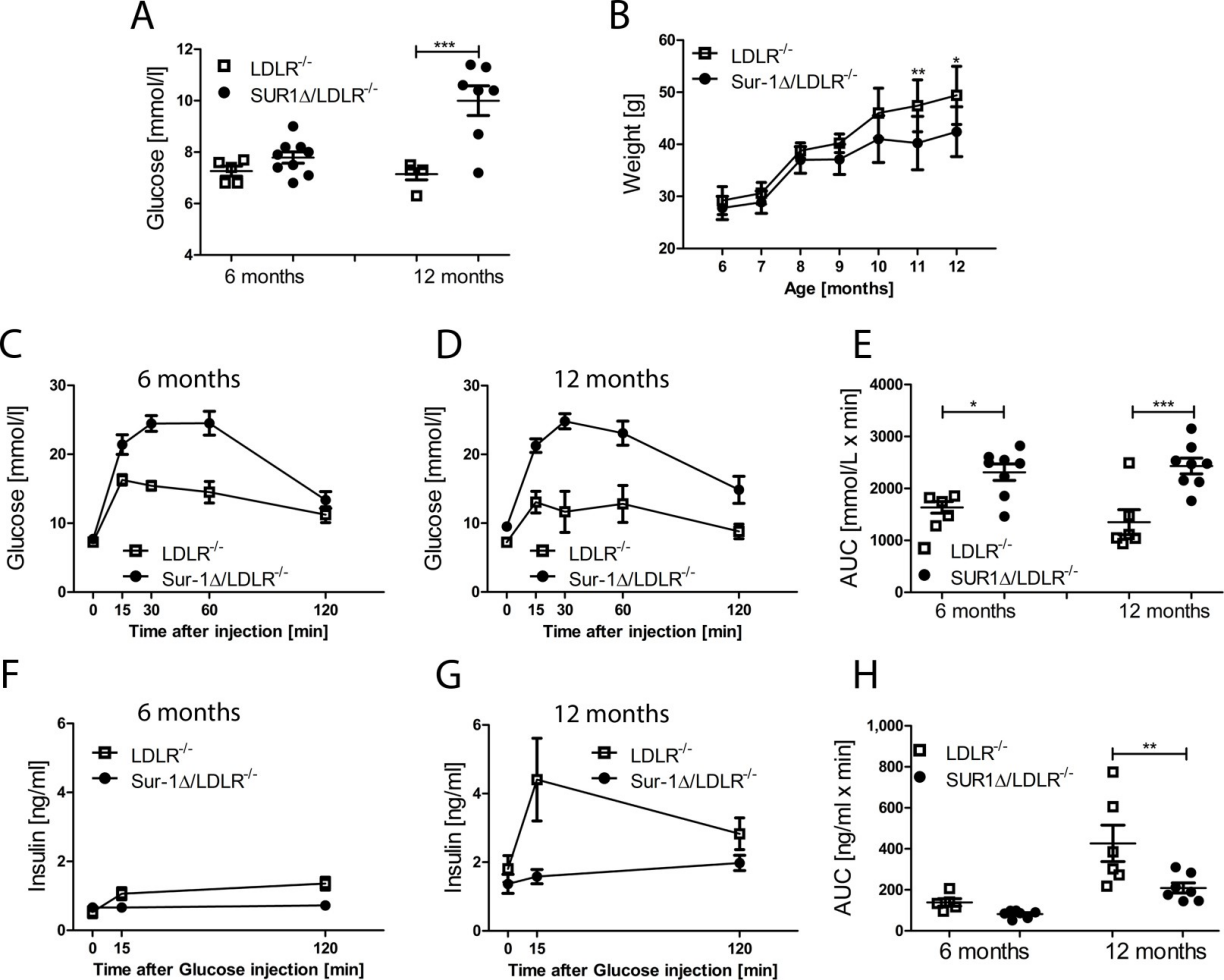
SUR1Δ/LDLR^-/-^ mice have increased fasting glucose levels and impaired glucose tolerance with decreased insulin secretion. (A) Fasting glucose levels of SUR1Δ/LDLR^-/-^ (n = 7–9) and LDLR^-/-^ (n = 5) mice at the age of 6 and 12 months, (B) Weight changes during WD of SUR1Δ/LDLR^-/-^ (n = 4–7) mice compared to their LDLR^-/-^ (n = 7) controls, (C and D) IPGTT at 6 and 12 months of age, (E) AUC during IPGTT of SUR1Δ/LDLR^-/-^ (n = 8) and LDLR^-/-^ (n = 5) mice, (F and G) Insulin secretion during IPGTT at the age of 6 and 12 months, (H) AUC of insulin secretion of SUR1Δ/LDLR^-/-^ (n = 5–6) compared to LDLR^-/-^ (n = 7–8) mice. Data are shown as mean ± SEM. For weight analysis two-way ANOVA, followed by Bonferroni-test was used. Otherwise, student’s t-test was applied for statistical analysis. * p < 0.05, ** p <0.01, *** p < 0.001.

In SUR1Δ/LDLR^-/-^ mice, fasting blood glucose was increased after 6 months WD compared to LDLR^-/-^ mice (Fig 4A). However, SUR1Δ/LDLR^-/-^ mice did not gain as much weight as their LDLR^-/-^ controls on the WD, and by the age of 11 months SUR1Δ/LDLR^-/-^ were significantly leaner than LDLR^-/-^ mice (Fig 4B). IPGTT indicated that both 6 and 12 months old SUR1Δ/LDLR^-/-^ mice were significantly more glucose intolerant when compared to LDLR^-/-^ mice (Fig 4C–4E). While insulin secretion during IPGTT is not changed between SUR1Δ/LDLR^-/-^ and LDLR^-/-^ mice at the age of 6 months, SUR1Δ/LDLR^-/-^ mice fed with an additional 6 months of WD had significantly reduced insulin secretion when compared to LDLR^-/-^ mice (Fig 4F–4H). No significant changes in insulin sensitivity could be detected between the groups during IPITT (S1 Fig).

### Enhanced expression of NLRP3 inflammasome and IL-1β in the aorta and macrophages of SUR1-E1506K mutated LDLR^-/-^ mice

**Fig 5:**
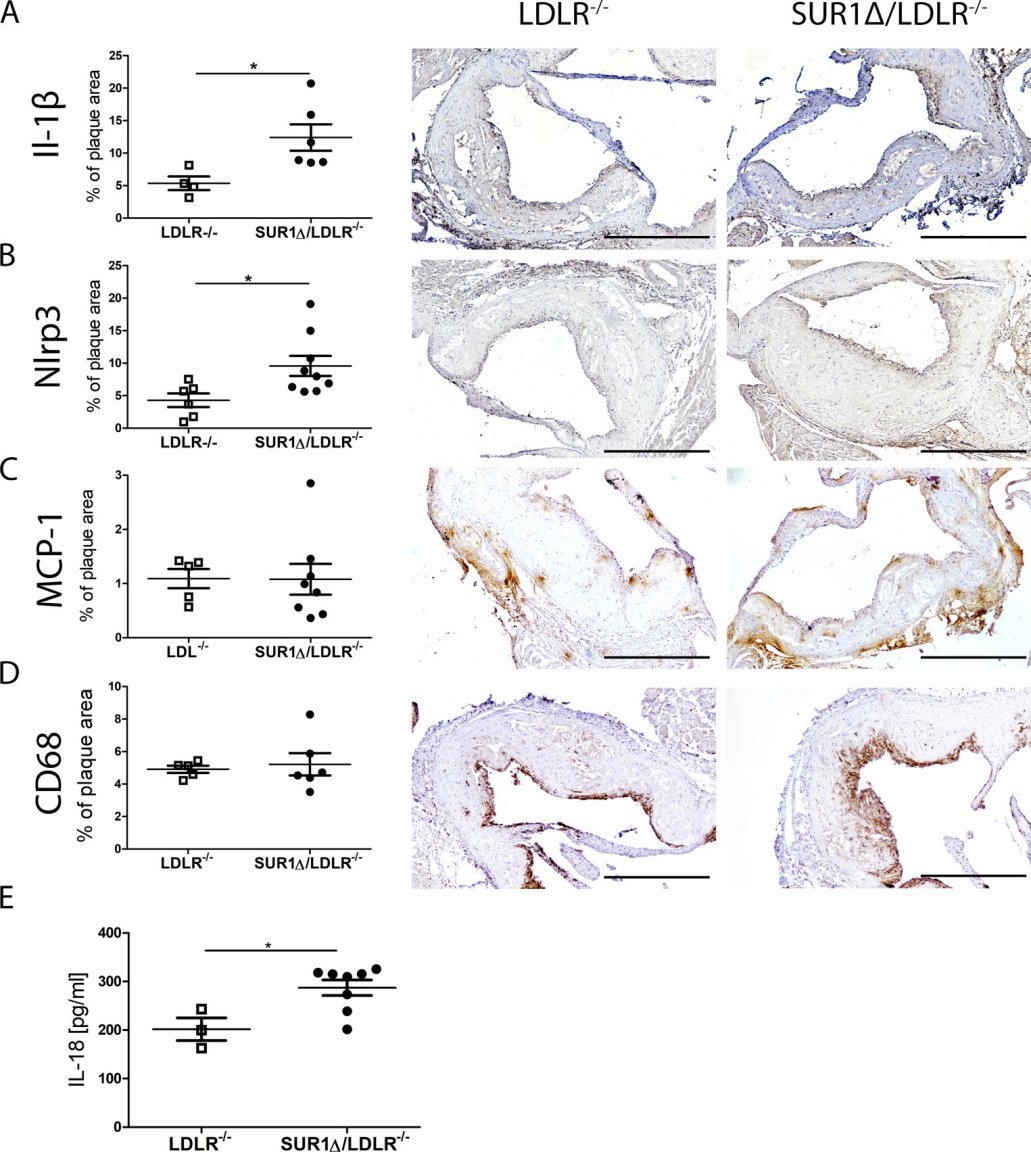
Enhanced NLRP3 inflammasome and IL-1β expression in aortic valves and increased IL-18 plasma levels in SUR1Δ/LDLR^-/-^ and LDLR^-/-^ mice. In immunohistological staining SUR1Δ/LDLR^-/-^ mice (n = 6–9) showed increased levels of (A) IL-1β and (B) NLRP3 in aortic plaques when compared to LDLR^-/-^ mice (n = 4–6). No changes were detected between SUR1Δ/LDLR^-/-^ (n = 8) and LDLR^-/-^ mice (n = 5–6) in (C) MCP-1 and (D) CD68 positive staining area in aortic plaques. Scale bars: 500μm. (E) IL-18 plasma levels were increased in SUR1Δ/LDLR^-/-^ mice (n = 3) in comparison to LDLR^-/-^ mice (n = 8). Results are shown as mean ± SEM and Student’s t-test was used for statistical analysis. * p < 0.05.

Enhanced arterial inflammation is one of the key driving factors of atherosclerosis in diabetes. Therefore, we investigated arterial inflammation of pro-inflammatory cytokines by histology in aortic root plaques. Interestingly, immunohistological staining from aortic plaques revealed that SUR1Δ/LDLR^-/-^ mice at age of 12 months have significantly increased plaque expression of IL-1β (Fig 5A) and increased positive staining area of NLRP3 compared to LDLR^-/-^ mice (Fig 5B). But no changes were detected with two different macrophage activity markers, MCP-1 and CD68 in aortic plaques (Fig 5C and 5D). In addition, plasma levels of IL-18 were significantly increased in SUR1Δ/LDLR^-/-^ mice compared to LDLR^-/-^ mice after WD at age of 12 months (Fig 5E).

**Fig 6:**
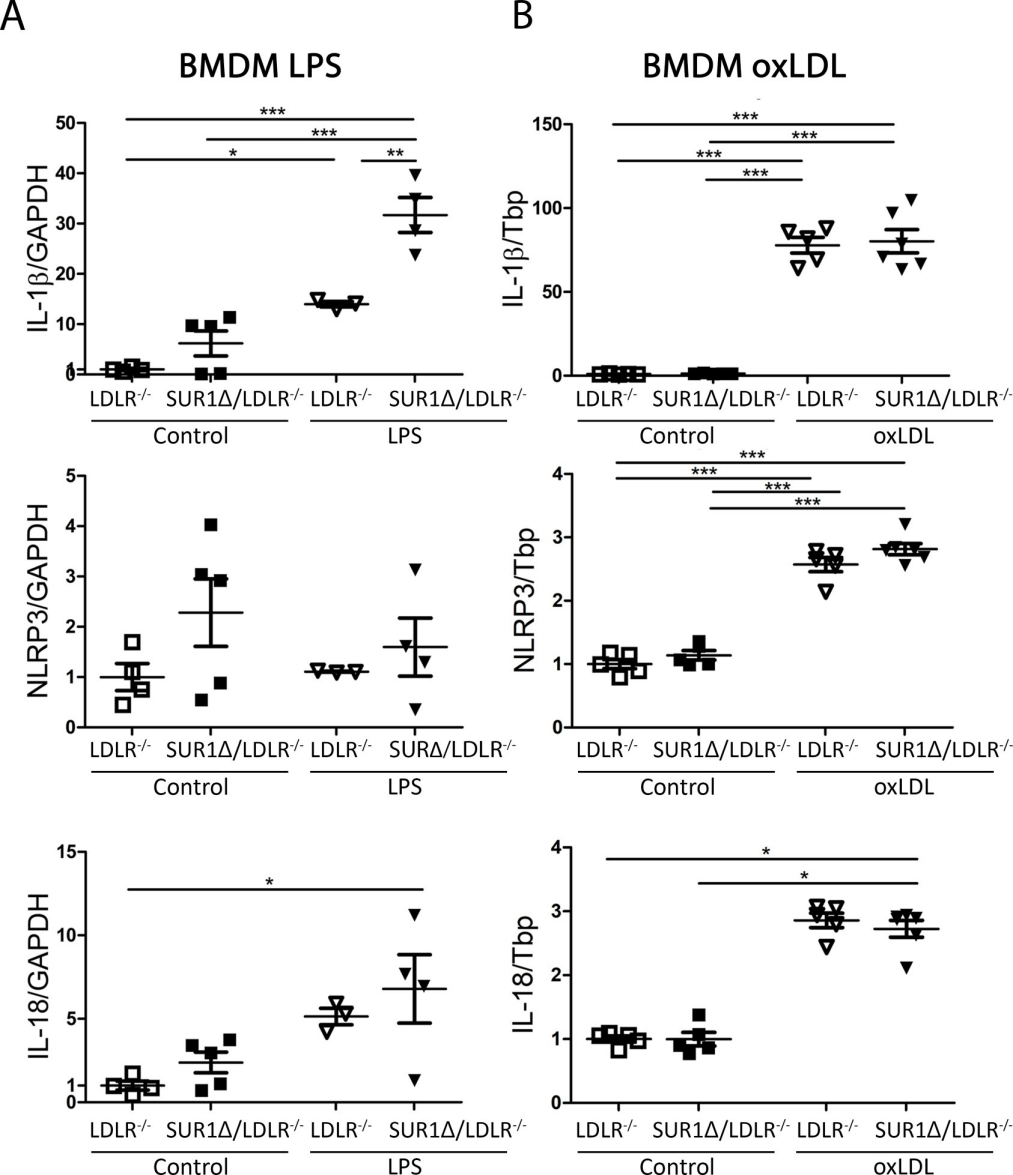
Enhanced mRNA expression of IL-1β s after LPS treatment in SUR1Δ/LDLR^-/-^ and LDLR^-/-^ BMDMs. (A) mRNA expression of IL-1β, NLRP3 and IL-18 after LPS treatment (n = 3–5). The expression was normalized with GAPDH. (B) mRNA expression of IL-1β, NLRP3 and IL-18 after oxLDL (50μg/ml) treatment (n = 5–6). The expression was normalized to Tbp. Results are shown as mean ± SEM. One-way ANOVA, followed by Bonferroni-test was used for statistical analysis * p < 0.05, ** p <0.01, *** p < 0.001.

BMDMss of SUR1Δ/LDLR^-/-^ mice showed increased IL-18 and IL-1β mRNA expression after LPS treatment compared to LDLR^-/-^ macrophages but no changes were seen in NLRP3 mRNA expression (Fig 6A). OxLDL treatment of BMDM led to increased levels of Nlrp3, IL-18 and Il-1 β when compared to nontreated macrophages. However, no difference between the strains could be seen (Fig 6B).

## Discussion

In type 2 diabetes, increased fasting glucose and impaired glucose tolerance promote the progression of atherosclerosis via several mechanisms and elevate the risk of cardiovascular complications independently of other conventional risk factors [4, 12]. It has been reported that hyperglycemia correlates with an increased area of early fatty streaks in mice, and early glycemic control possibly reduces the development of advanced plaques [21–23]. Nevertheless, the role and importance of hyperglycemia in atherogenesis and plaque vulnerability in type 2 diabetic patients have been controversial, as results for and against beneficial effects of glucose lowering have been reported in clinical and epidemiological studies [24–26]. Therefore, further studies are required to clarify the mechanisms on how diabetes affects atherogenesis. In this study, we show that loss-of-function mutation SUR1-E1506K in LDLR^-/-^ mice impairs glucose tolerance and elevates fasting glucose without affecting blood lipid levels, leading to a more vulnerable plaque phenotype possibly via enhanced arterial inflammation.

Backcrossing of SUR1 mice to LDLR^-/-^ background provides a promising tool to investigate the mechanisms of atherogenesis and its complications in diabetic conditions. The E1506K knock-in mutation is reported to increase insulin secretion and to lower blood glucose in early life in C57Bl/6J mice [16]. Similarly to patients having that mutation, the insulin secretion is reduced with aging in mice [16]. It has been proposed that chronic exposure to high glucose or elevated intracellular calcium by SUR1 mutation downregulates insulin gene transcription and thereby insulin secretion [16]. However, the exact mechanism is not known. In almost all mouse models of diabetes, plasma lipid levels are elevated by the induction of diabetes. This does usually not occur in humans and thereby complicates the differentiation between the effects of diabetes and hyperlipidemia on atherogenesis [27]. In LDLR^-/-^ mice, SUR1-E1506K mutation induces increased fasting glucose levels and impaired glucose tolerance without changes in plasma lipid levels. However, the moderate increase in blood glucose compared to LDLR^-/-^ mice indicates a pre-diabetic phenotype. Furthermore, most of the hypercholesterolemic mouse models currently used are modeling a fast progression of atherosclerosis [28], and they do not show vulnerable plaque phenotype leading to myocardial infarction and stroke as seen in humans [29].

Hyperglycemia has several proatherogenic effects as it induces vascular oxidative stress and inflammation, alters vascular function, permeability, cell growth and apoptosis [30]. In addition, hyperglycemia increases the formation of advanced glycated end products (AGEs) and promotes proatherogenic modifications in lipoproteins. In this study, we showed that the pre-diabetic condition did not have an effect on atherosclerotic plaque size in the aortic root or in the brachiocephalic artery. This is most likely due to equal plasma total cholesterol, triglyceride and free fatty acid levels between the groups. These findings demonstrate that hyperglycemia does not independently increase the area of advanced plaques, but more likely plays a role in plaque maturation and stability. This is supported by our findings that impaired glucose metabolism in SUR1Δ/LDLR^-/-^ mice increased the plaque necrotic area and reduced the number of smooth muscle cells, indicating the unfavorable plaque morphology when compared to LDLR^-/-^ mice. This was also confirmed by increased plaque vulnerability index and the reduced fibrous cap thickness in SUR1Δ/LDLR^-/-^ mice compared to LDLR^-/-^ mice. Although the plaques were more mature and vulnerable, no intraplaque hemorrhages were detected. The occurrence of spontaneous intraplaque hemorrhage is very rare in mice without surgical interventions, and currently, only ApoE knock out mice with mutated *Fbn1*gene have been reported to have spontaneous intraplaque hemorrhages [29, 31]. We could, however, show that the number of adventitial microvessels, another hallmark of unstable plaques, was increased in SUR1Δ/LDLR^-/-^ mice compared to LDLR^-/-^ mice. This is supported by the report that plaque progression correlates with an increase in adventitial microvessels [10].

Local vascular inflammation plays a central role in the progression of rupture-prone advanced plaques. Hyperglycemia and vascular inflammation are both known to increase leukocyte and monocyte adhesion and accumulation in atherosclerotic plaques [12, 26, 32]. In this study, we did not see differences in plaque macrophage content in aortic root, as plaque phenotype was already highly necrotic and acellular in both groups. Nevertheless, increased cytokine IL-1β and inflammasome NLRP3 expression in aortic sections indicated increased vascular pro-inflammatory stimulus in SUR1Δ/LDLR^-/-^ mice in comparison to LDLR^-/-^ mice. Several studies have reported the crucial role of NLRP3 in atherogenesis and plaque destabilization in the association with diabetes [33, 34]. In the present study, the increased NLRP3 expression in SUR1Δ/LDLR^-/-^ mice is also supported by increased IL-18 plasma levels when compared to LDLR^-/-^ mice. This is in line with the report where the activation of NLRP3 led to increased secretion of IL-18 in human carotid atherosclerotic plaques associating with plaque vulnerability [35]. Interestingly, the expression of IL-1β and IL-18 were increased in BMDMs after LPS, but not after oxLDL treatment in SUR1Δ/LDLR^-/-^ mice in comparison to LDLR^-/-^ macrophages. These results indicate that both diabetic condition and possibly SUR1-E1506K mutation itself activates NLRP3 inflammasome and increases the expression of IL-1β and IL-18 both in the vascular wall and in macrophages *in vitro*. Our results are supported by other reports, where NLRP3 is activated by hyperglycemic conditions and involved in type 2 diabetes [36].

In summary, our findings suggest that hyperglycemia and glucose intolerance caused by the SUR1-E1506K mutation in LDLR^-/-^ mice promote a vulnerable plaque phenotype, which is possibly due to increased vascular inflammation and NLRP3 inflammasome activation.

## Supporting information

S1 Fig SUR1Δ/LDLR^-/-^ do not have impaired insulin sensitivity nor are fasted insulin levels changed when compared with LDLR^-/-^ mice. Measurement of IPITT at (A) the age of 6 months (B) the age of 12 months (C) AUC during IPITT in SUR1Δ/LDLR^-/-^ (n = 7) and LDLR^-/-^ (n = 6–7) mice. Data are shown as mean ± SEM and Student’s t-test was used for statistical analysis.
(TIF)

S1 Data (XLSX)
