# Supplementary figures and images for: SUR1-E1506K mutation impairs glucose tolerance and promotes vulnerable atherosclerotic plaque phenotype in hypercholesterolemic mice
Source: PLoS One. 2021 Nov 12;16(11):e0258408. doi: 10.1371/journal.pone.0258408 (PMC8589160; doi:10.1371/journal.pone.0258408)

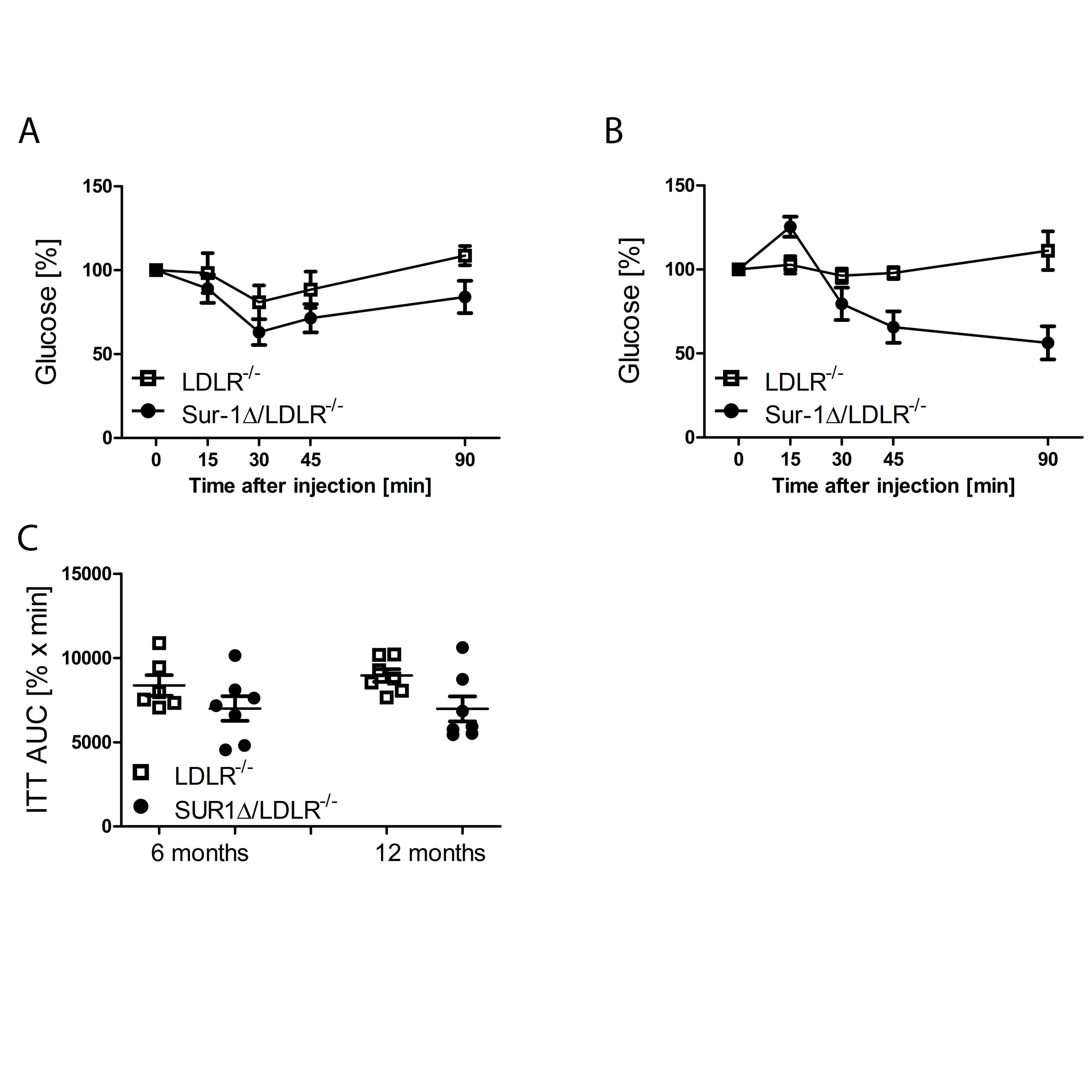

Supplement: S1 Fig — Measurement of IPITT at (A) the age of 6 months (B) the age of 12 months (C) AUC during IPITT in SUR1Δ/LDLR-/- (n = 7) and LDLR-/- (n = 6–7) mice. Data are shown as mean ± SEM and Student’s t-test was used for statistical analysis. (TIF) [file pone.0258408.s001.tif]
